# The Effect of Adding Remifentanil to Thiopental for Anaesthesia Induction on the Success of Classic Laryngeal Mask Airway Insertion: A Randomised Double-Blind Clinical Trial †

**DOI:** 10.3390/ph18050654

**Published:** 2025-04-29

**Authors:** Mensure Çakırgöz, İsmail Demirel, Mert Akan, Ömürhan Saraç, Ergin Alaygut, Aysun Afife Kar, Oğuzhan Demirel, Emre Karagöz

**Affiliations:** 1 Intensive Care Unit, Department of Anesthesiology and Reanimation, Izmir City Hospital, University of Health Sciences, Izmir 35540, Turkey; dromurhansarac@hotmail.com (Ö.S.); aaysunkar@hotmail.com (A.A.K.); dr.emrekaragoz@gmail.com (E.K.); 2 Intensive Care Unit, Department of Anesthesiology and Reanimation, Firat University School of Medicine Hospital, Elazig 23119, Turkey; ismaildemirel23@gmail.com; 3 Intensive Care Unit, Department of Anesthesiology and Reanimation, Izmir Acibadem Kent Hospital, Izmir 35620, Turkey; mertakan@hotmail.com; 4 Intensive Care Unit, Department of Anesthesiology and Reanimation, Izmir Tepecik Training and Research Hospital, University of Health Sciences, Izmir 35180, Turkey; ealaygut@yahoo.com; 5 Department of Anesthesiology and Reanimation, Ankara University Ibni Sina Hospital, Ankara 06052, Turkey; demireloguzhan1@gmail.com

**Keywords:** remifentanil, thiopental, laryngeal mask airway, anaesthetic induction

## Abstract

**Background**: Remifentanil, an ultra-short-acting µ-receptor agonist, is used with propofol for optimal laryngeal mask airway (LMA) insertion. However, no studies have assessed its effects when combined with thiopental on LMA conditions. The combined use of thiopental and remifentanil may offer advantages, such as enhanced cardiovascular and respiratory stability. This study aims to compare the administration of thiopental with different doses of remifentanil to assess their combined effects on LMA insertion conditions and success in a prospective, randomised double-blind study. **Method**: A total of 100 ASA I–II patients (18–65 years), including both male and female participants, were randomly assigned to four remifentanil dose groups (0.5–3 µg.kg^−1^). Induction involved thiopental (5 mg.kg^−1^) after remifentanil. LMA insertion conditions were evaluated using a six-variable scale. Systolic arterial pressure (SAP), diastolic arterial pressure (DAP), mean arterial pressure (MAP), heart rate (HR), and bispectral index monitor (BIS) values, as well as apnoea duration, eyelash reflex loss time, and insertion attempts, were recorded at baseline, before insertion, and at 5 min post-insertion. **Results**: Time to eyelash reflex loss and LMA insertion were shorter in Groups III and IV than in Groups I and II (*p* < 0.001). Apnoea duration was longest in Group IV, followed by Group III (*p* < 0.001). Groups III and IV had significantly better LMA placement, mouth opening, and ease of insertion (*p* < 0.05). Coughing and gagging were highest in Group I (*p* < 0.001). SAP, MAP, HR, and DAP were significantly lower in Group IV at various time points (*p* < 0.05). HR was significantly higher in Group I compared to Groups II and III at multiple time points (*p* < 0.05). **Conclusions**: The administration of 5 mg.kg^−1^ thiopental with 2 μg.kg^−1^ remifentanil has been found to provide a stable haemodynamic response and 96% excellent or satisfactory laryngeal mask insertion conditions without increasing the duration of apnoea.

## 1. Introduction

The laryngeal mask is a non-invasive supraglottic airway device (SAD) inserted blindly into the hypopharynx to close around the larynx [1,2]. It was designed in 1981 by Archie Brain to avoid common problems in airway management. It became available commercially and clinically in 1988 [1].

The laryngeal mask offers several clinical advantages, including reduced pharyngolaryngeal morbidity, improved haemodynamic stability, and faster insertion times compared to endotracheal intubation. It is preferred in short-duration surgeries due to being less invasive than intubation, plays a key role in difficult airway management, facilitates fibreoptic intubation, and is particularly beneficial in patients with cardiovascular instability or difficult intubation conditions [2,3].

Successful placement of the laryngeal mask requires an adequate depth of anaesthesia for appropriate mouth opening and suppression of upper airway reflexes such that the mask will be tolerated in the hypopharynx, without undesirable responses, such as coughing, gagging, or laryngospasm [4,5,6,7]. Propofol is superior to thiopental and other induction agents due to its depressant effects on laryngeal responses and upper airway reflexes, and, thus, it is the most frequently chosen induction agent [6,7,8,9,10]. However, routine use has been questioned due to injection pain, expense, serious cardiorespiratory depression, and increased apnoea risk linked to high-dose requirements when used alone [4,5,6,8,11]. Thiopental is less costly compared to propofol, is easy to access, does not cause injection pain, causes less haemodynamic depression, and has a shorter apnoea duration; however, alone, it does not provide sufficient conditions for LMA insertion [4,10,12]. For this reason, to improve LMA insertion conditions, several agents are used as adjuvants to thiopental, including the opioids fentanyl [10], alfentanil [7], and butorphanol [4]; topical lidocaine; intravenous midazolam [12,13]; low-dose succinylcholine [8]; and low-dose atracurium [14].

Remifentanil is an ultra-short-acting selective μ agonist with a half-life of 3.8–8.3 min that is increasingly frequently chosen for routine anaesthesia administration; it is rapidly and “organ-independently” metabolised by non-specific blood and tissue esterase [15]. Remifentanil has been successfully used with propofol or thiopental for tracheal intubation without the use of a muscle relaxant, which requires deeper anaesthesia planning compared to LMA insertion [16,17,18,19,20,21,22,23,24]. However, our literature review did not encounter any study assessing the effects of the combination of remifentanil with thiopental on LMA insertion conditions. The aim of our study was to examine the effect of different doses of remifentanil (0.5, 1, 2, or 3 μg.kg^−1^) with 5 mg.kg^−1^ thiopental induction on LMA insertion conditions and success, with the hypothesis that the administration of remifentanil at 3 μg.kg^−1^ in combination with thiopental will result in successful LMA insertion. We also aimed to determine the optimal remifentanil dose for successful LMA insertion in a prospective, randomised, and double-blind clinical study.

## 2. Results

The demographic data, including age, gender, and weight, and the duration of LMA use were similar across the four groups (*p* > 0.05) (Table 1). The time to loss of eyelash reflex and LMA insertion time were significantly shorter in Group III and Group IV compared to Group I and Group II (*p* < 0.0001). Apnoea duration in Group IV was significantly longer (*p* < 0.0001) than in the other groups, and in Group III, it was significantly longer than in Group II (*p* < 0.01) (Table 2).

As shown in Table 3, the rate of excellent LMA placement conditions and full mouth opening was significantly higher in Groups III and IV compared to Groups I and II (*p* < 0.001, *p* < 0.01). Additionally, the ease of LMA placement was significantly higher in Groups III and IX compared to Group I (*p* < 0.05). The rate of coughing and gagging was significantly higher in Group I compared to the other three groups, and in Group II compared to Groups III and IV (*p* < 0.05, *p* < 0.001). No statistically significant differences were found between the groups in terms of swallowing, movement, number of attempts, or incidence of laryngospasm (*p* > 0.05).

Baseline SAB, DAB, HR, and MAP values did not differ significantly among the groups (*p* > 0.05). At 1 min before LMA insertion and all time points after LMA insertion, SAB and MAP values were lower in Group IV than in the other groups, and HR and DAP values were lower in Group IV than in Group I (*p* < 0.0001, *p* < 0.01). DAB was significantly lower in Group IV than in Groups II and III at 1, 2, and 3 min after LMA insertion (*p* < 0.01). HR values in Group I were significantly higher than in Group III at 1, 2, and 3 min after LMA insertion and significantly higher than in Group II at 4 and 5 min (*p* < 0.01). Ephedrine was administered due to hypotension in one patient in Group III and three patients in Group IX (Table 4). There were no significant differences between the four groups in terms of the presence of blood on the mask, sore throat, or dysphagia upon discharge from the recovery unit (*p* > 0.05) (Table 5).

## 3. Discussion

Remifentanil, with its rapid onset and short duration, is ideal for outpatient procedures such as LMA insertion and tracheal intubation, providing rapid recovery. It has been associated with significant hypokinetic cardiovascular events, such as decreased HR and/or arterial pressure, at a higher rate than other opioids [25,26,27,28,29]; additionally, bradyarrhythmia, frequently accompanying remifentanil in clinical practice, has been shown to be associated with a temporary increase in parasympathetic tonus compared to sympathetic tonus [25,26]. Therefore, it is recommended to administer the pure anticholinergic agent atropine to induce pre-emptive vagolysis [15,17,19,24,25,26,30]. As a result, the risk of bradycardia and hypotension increases when propofol is administered with remifentanil [19,25,28,29,30,31]. However, in tracheal intubation without the use of a muscle relaxant, it has been reported that thiopental likely causes less vasodilation and suppression of baroreflex responses compared to propofol, resulting in a more stable haemodynamic response when used in combination with remifentanil, without leading to an increase in apnoea duration [19,20,21,29,32]. This study is the first to evaluate the effect of thiopental, along with remifentanil administration, on LMA insertion conditions. The results of our study show that 5 mg.kg^−1^ thiopental with pre-induction bolus of 2 μg.kg^−1^ remifentanil provides an acceptable haemodynamic response without increased apnoea duration and 96% perfect or satisfactory conditions for laryngeal mask insertion in healthy patients with suitable airway anatomy given premedication [33].

In studies investigating which induction technique provides optimum conditions for successful LMA insertion without increasing apnoea duration and cardiovascular depression, thiopental has been used alone or in combination with several agents. In the literature, according to the references we can access, five different induction techniques using 5 mg.kg^−1^ thiopental, in addition to topical lidocaine [12], opioids [4,13], benzodiazepines [4,13], and low-dose muscle relaxants alone [8,14] or in combination [4,8,12,13,14], provided perfect or satisfactory LMA insertion conditions at similar rates to those found in our study. Cook et al. [12] used thiopental with pre-induction IV 1 μg.kg^−1^ fentanyl, along with 40 mg topical lidocaine sprayed on the posterior pharyngeal wall. Though they did not use a similar assessment scale as in our study, they reported similar rates of perfect laryngeal mask insertion conditions (73% and 72%, respectively). However, when compared with 40 mg topical lidocaine administration, lower rates of laryngospasm (3.3% vs. 0%), poor/unacceptable LMA insertion conditions (13% vs. 4%), and coughing/gagging (30% vs. 4%) were found in our study. Compared with fentanyl administered with topical lidocaine, the administration of the more potent and short-effect opioid of remifentanil caused greater suppression of airway reflexes. Though not clinically significant, the mean apnoea duration was nearly 2.5 times higher (96 s and 232 s, respectively), which may be associated with the higher proportion of female patients in our study and our administration of premedication (midazolam). The higher laryngospasm and coughing/gagging rates in the study by Cook et al. [12] may be related to the higher proportion of male patients and the insertion of the mask 15 s after anaesthesia induction. In our study, though MAP and HR decreased by 12% and 8% after induction of anaesthesia, the variation with topical lidocaine was less than 10%, with similar hypotension incidence despite different thresholds. Though topical lidocaine may be a cost-effective alternative with short apnoea duration and stable haemodynamics, it poses an aspiration risk due to potential vomiting or regurgitation, particularly during awakening from anaesthesia [34].

Similar rates of perfect or satisfactory LMA insertion conditions (96%, 95%, and 100%, respectively) were reported by Yoshino et al. [8] with low-dose succinylcholine (0.5 mg.kg^−1^) after induction with thiopental; by Koh et al. [14], with priming atracurium (0.1 mg.kg^−1^) 1 min before induction with thiopental; and in our study. In the study by Yoshino et al. [8] comparing thiopental alone with the co-administration of succinylcholine (0.25 mg.kg^−1^, 0.5 mg.kg^−1^), despite no significant difference in full mouth-opening rates with increasing succinylcholine doses (10%, 15%, and 35%, respectively), the extension of apnoea duration (84.7 s, 194.9 s, and 234.2 s, respectively), and the reduction in coughing and gagging (85%, 65%, and 15%, respectively), an increase was reported for perfect or satisfaction laryngeal mask insertion conditions (40%, 60%, and 95%, respectively). Similarly, Stoneham et al. [5] reported that IV lidocaine (1.5 mg.kg^−1^) administration before propofol caused an increase in satisfactory laryngeal mask insertion conditions linked to the suppression of laryngeal reflexes such as coughing and laryngospasm, along with a reduced propofol induction dose and reduced incidence of gagging without causing a significant increase in mouth-opening degree. Brimacombe [35] concluded that there was no significant difference in terms of ease of LMA insertion between administration of 1 μg.kg^−1^ fentanyl-2.5 mg.kg^−1^ propofol and 4–6 mg.kg^−1^ thiopental-1 mg.kg^−1^ succinylcholine in a follow-up study. For this reason, though an improvement in LMA insertion conditions was provided, there was no advantage to administering a neuromuscular blocker agent compared to the condition of administering a sufficient dose of an induction agent. The results of our study (using 2 μg.kg^−1^ remifentanil) are consistent with those in studies by Yoshino et al. [8] and Koh et al. [14]. Despite variations in full jaw-opening rates (84%, 35%, and 93%, respectively), there was a lower rate of coughing/gagging (4%, 15%, and 13%, respectively) with no cases of laryngospasm and perfect or satisfactory LMA insertion conditions across all three applications. This suggests that the main cause of failed LMA insertion is residual upper respiratory tract reflexes that may cause gagging, coughing, and/or laryngospasm. Additionally, even at low doses, succinylcholine use during induction raises the risk of malignant hyperthermia, arrhythmia, anaphylaxis, and myalgia, while neuromuscular blocker priming may cause discomfort, hypoventilation, aspiration, and reduced pulmonary function, particularly in elderly patients [8,14].

Finally, in our study, the administration of 2 μg.kg^−1^ remifentanil provided similar rates of perfect or satisfactory LMA insertion conditions as those found in two other studies (96% and 98%, respectively) by Bapat et al. [13], who used IV 1 μg.kg^−1^ fentanyl and 0.1 mg.kg ^−1^ midazolam before induction with 5 mg.kg^−1^ thiopental, and by Chari et al. [4], who used 30 mg.kg^−1^ butorphanol. When the results of our study are compared with those of Bapat et al. and Chari et al., though our full mouth-opening rates were lower (84%, 94%, 92%), there were lower rates of coughing/gagging (4%, 10%, and 13.5%), movement (4%, 10%, and 11.5%), and swallowing (4%, 14%, and 13.5%), and no patient developed laryngospasm (0%, 6%, and 11.5%), showing that remifentanil administration caused higher rates of suppression of upper airway reflexes. Additionally, the administration of midazolam and butorphanol caused high postoperative first-hour sedation scores. This may be associated with the high mean age in both studies and with the fact that the population in the study by Chari et al. comprised healthy female patients. Though discharge criteria were reported not to be affected, there may be a risk of extension of recovery duration. With similar rates of perfect or satisfactory LMA insertion conditions compared to these five administrations, we believe that 2 μg.kg^−1^ remifentanil administration before induction suppresses airway reflexes more without causing a clinically significant increase in apnoea duration and will be a more suitable alternative to short-duration and/or outpatient surgical interventions.

The laryngeal mask can be inserted blindly without a laryngoscope, so it is less invasive and causes less stimulation compared to endotracheal intubation [1]. This situation means that sufficient anaesthesia conditions may be provided with lower doses of remifentanil for laryngeal mask insertion compared to tracheal intubation. In our study, we determined our remifentanil dose interval based on half of the lowest dose [23] and half of the highest dose [22] of remifentanil used with thiopental without a muscle relaxant for intubation.

Remifentanil suppresses laryngeal reflexes more than other opioids, enhances insertion conditions without muscle relaxants, and reduces the hypnotic requirements for induction and linked haemodynamic instability [15,18,20,28,29,36]. Durmuş et al. [16] and Mohammadreza et al. [20] administered 0.03 mg.kg^−1^ IV midazolam 10–15 min before induction and then 5 mg.kg^−1^ thiopental with 2, 3, and 4 μg.kg^−1^ remifentanil before induction and reported rates of perfect intubation of 6–5%, 49–45%, and 89–95%, respectively, with increasing doses. Contrary to the previous two studies, Bouvet et al. [22] performed a dose–response study to determine the intubation effective dose (IED) of remifentanil in the 3 to 6 μg.kg^−1^ interval with 5 mg.kg^−1^ thiopental induction to ensure 95% perfect intubation conditions (IED95) for healthy female patients undergoing elective surgery with premedication. They found perfect intubation conditions at a rate of 35% in the 4 μg.kg^−1^ remifentanil group and 76% in the 6 μg.kg^−1^ remifentanil group. Additionally, despite not observing a statistically significant difference between the groups during the study, ephedrine needs increased with increasing dose, along with a mean 26–28% fall in MAP, and a significant fall in HR was reported in the 6 μg.kg^−1^ remifentanil group. When remifentanil was administered at doses higher than 6 μg.kg^−1^, the haemodynamic response could not be assessed, and when 95% of patients had perfect intubation conditions, the remifentanil dose was reported to be more than 7 μg.kg^−1^. In our study, similar to the study by Bouvet et al. [22], with the increase in haemodynamic depression with increasing dose, we did not identify a significant difference in terms of perfect LMA insertion conditions with the administration of 2 or 3 μg.kg^−1^ remifentanil (72% and 80%, respectively). With the current findings, for LMA insertion with a much lower intensity stimulus compared to tracheal intubation, we believe that there is a probable risk of severe haemodynamic depression at higher doses, while 95% rates of perfection insertion conditions may be reached.

Rapid administration of high doses of remifentanil (more than 1 µg.kg^−1^) may cause side effects like bradycardia and muscle rigidity [15,20,22,30]. To avoid this, slow infusion or bolus over at least 30 s is recommended [15,21]. Additionally, pretreatment with benzodiazepines and co-administration with a hypnotic agent may protect against opioid-induced rigidity [15,16,18,19,20,21,22,30,37]. In our study, all patients could be easily ventilated with a mask. Our results, consistent with the results of previous studies with similar drug combinations and dosages [15,16,18,20,21,22,30,37], indicate that our pretreatment with benzodiazepines and slow administration of remifentanil over 60 s with a hypnotic agent administered for 30 s may have contributed to preventing rigidity.

Remifentanil reduces the haemodynamic response to tracheal intubation and LMA insertion [16,18,20,21,22,23]. In our study, SAP, MAP, DAP, and HR were significantly lower in Groups II, III, and IV after induction and LMA insertion compared to basal values, while the values in Group IV were significantly lower compared to Groups I, II, and III (*p* < 0.05) [16,20,22,23]. Additionally, consistent with previous studies, there was an increase in acceptable LMA insertion conditions and haemodynamic depression with increasing remifentanil dose. Administration of 3 μg.kg^−1^ remifentanil resulted in 21%, 24%, 22%, and 13% falls in SAP, DAP, MAP, and HR after induction, respectively. Three patients in Group III and one patient in Group II were given ephedrine. In studies using similar agents and doses of remifentanil for induction before tracheal intubation without muscle relaxants, Mohammadreza et al. [20] and Durmuş et al. [16] reported that post-induction MAP with 3 μg.kg^−1^ remifentanil decreased by 11% and 15%, respectively. Possible reasons why the same dose of remifentanil may have caused less haemodynamic depression in their studies include that midazolam, which takes 4 min to reach peak efficacy, was administered 5 to 10 min before induction; remifentanil was administered over a longer time (90 s); and Durmuş et al. also administered atropine before induction. Although we did not administer an anticholinergic agent before induction in our study, consistent with previous studies, heart rate (HR) continuously decreased before induction and after LMA insertion due to increasing doses of remifentanil; however, this reduction did not require any treatment [19,22]. This may be related to our slow administration of remifentanil during induction and the fact that all of our patients were ASA I or II, which may have contributed to the observed response.

The dose required for induction with thiopental reduces with age and is less for women than for men. Avram et al. [38] investigated the effect of age, sex, body weight, fat-free body mass, and cardiac output on thiopental dose requirements. They found that age, body weight, and fat-free body mass were the most significant factors for achieving a loss of voluntary motor power or burst suppression on EEG. Sex and cardiac output had smaller contributions to predicting the dose required for both clinical and EEG endpoints. In our study, anaesthesia depth was monitored with BIS and the target hypnotic level before LMA insertion was identified as below 40. For this reason, with the fixed induction dose administration, we believe that we may have prevented different LMA insertion conditions that emerge as a result of variable anaesthesia depth and differences in the thiopental dose requirements linked to both age and patient-specific variables.

Wilhelm et al. [29] evaluated the effects of remifentanil and fentanyl on induction with propofol, thiopental, or etomidate. They found that remifentanil shortened the time to loss of eyelash reflex and improved induction quality when combined with these agents. In our study, the time to eyelash reflex loss with administration of the same dose of remifentanil (0.5 μg.kg^−1^) was shorter than the duration reported in the study by Wilhelm et al. (50.7 and 39.8 s, respectively). This may be related to the administration times of the drugs for induction, our administration of additional 0.03 mg.kg^−1^ IV midazolam, and the administration of remifentanil for infusion instead of bolus. Additionally, consistent with the study by Jeon et al. [17], we identified that the time to loss of eyelash reflex reduced with the increase in remifentanil dose.

Consistent with previous studies, with the increase in apnoea duration with increasing remifentanil dose in our study [17,23,39] and the improvement in LMA insertion conditions, we identified a reduction in LMA insertion duration [23,40]. The study by Jeon et al. [23], with a similar design to our study, had longer apnoea durations (276 and 612 s) with administration of 2 and 3 ug kg^−1^ remifentanil. We believe that the following factors contributed: firstly, the mean age in the study group being higher, and secondly, the administration of 50% nitrous oxide containing 2% sevoflurane with 60 s mask ventilation after induction. This has a very rapid effect due to the low blood gas solubility coefficient (0.67) and has been shown to have a synergic interaction with remifentanil in terms of sedation and analgesia.

Supraglottic airway devices cause lower pharyngolaryngeal morbidity and dysphonia compared to endotracheal intubation, despite variations in incidence [41,42]. The most probable cause of pharyngeal morbidity due to LMA insertion is trauma during insertion, influenced by several factors [43]. Throat pain, a common postoperative side effect, contributes to morbidity and dissatisfaction [41,42,43]. Studies comparing LMA-Classic™ and tracheal intubation showed higher throat pain incidence with intubation (39% vs. 17%) [43]. Throat pain linked to LMA insertion is multifactorial, affected by anaesthesia depth, neuromuscular blockers, insertion method, experience, mask size, cuff volume/pressure, duration, analgesia, N_2_O diffusion, and heated moisture exchanger [41,42]. Chui et al. [44] reported that increased doses of mivacurium, in addition to propofol, reduced the incidence of adverse reactions, increased the ease of LMA insertion, and caused the incidence of throat pain to fall from 53% to 24%. Chia et al. [41] compared propofol with thiopental and reported lower early pharyngeal morbidity incidence, including throat pain and dysphagia due to greater depression of laryngeal reflexes (in the second postoperative hour, throat pain was 24% vs. 13%, and dysphagia was 15% vs. 3% for thiopental and propofol administration, respectively). In our study, with the administration of increasing doses of remifentanil (0.5, 1, 2, and 3 μg.kg^−1^) before 5 mg.kg^−1^ thiopental, we identified reductions in the incidence of throat pain (31.8%, 25%, 16.6%, and 12%, respectively), dysphagia (27.2%, 20.8%, 8.3%, and 8%, respectively), and bleeding (22.7%, 16.7%, 8.3%, and 4%, respectively). Though there were significant differences between the groups, compatible with previous studies, we believe that the reduction in dysphagia and throat pain linked to the increase in remifentanil dose was associated with improvements in LMA insertion conditions, along with reductions in the number of attempts and airway trauma.

One of the limitations of our study is that the results are only valid for patients aged 18 to 65 years and classified as ASA I and II. Patients in ASA groups III or IV, especially with serious cardiac disease, may have different haemodynamic tolerances to the administration of remifentanil at doses higher than 2 μg.kg^−1^. We did not create a control group, as we did not consider it ethical due to the increased risk of airway trauma and inadequate ventilation, since administration of thiopental for induction alone may not sufficiently suppress pharyngolaryngeal reflexes. Future studies should explore the level of analgesia achievable with the remifentanil–thiopental combination. Additionally, trials should evaluate this co-induction in higher-risk cohorts and compare different SGA types in terms of insertion success, haemodynamic response, and postoperative airway comfort.

## 4. Materials and Methods

### 4.1. Study Subjects

This study included 100 patients in class I–II according to ASA physical status classification, aged 18–65 years, undergoing elective surgery, without requiring a muscle relaxant, with operation duration not exceeding 2 h, and with indications for LMA insertion. Patients with any neck or upper respiratory tract pathology, gastric content regurgitation–aspiration risk, or a history or probability of difficult airway conditions (Mallampati class 3–4, sternomental distance less than 12 cm, thyromental distance less than 6 cm, head extension less than 90 degrees, and mouth opening less than 1.5 cm); who were morbidly obese (Body Mass Index (BMI) > 40 kg/m^2^); who had a history of pulmonary disease; had an allergy to the study medications; had a history of alcohol and substance abuse; had a history of chronic sedative and opioid analgesic use; and had adrenocortical insufficiency, sore throat, dysphagia, and dysphonia were not included in the study.

### 4.2. Experimental Design

This prospective, randomised trial adopted a parallel-group design. Patients were randomly assigned to receive 0.5, 1, 2, or 3 µg.kg^−1^ remifentanil (Groups I, II, III, and IV, respectively, with *n* = 25 in each group). Randomisation was performed using a closed-envelope method to ensure the integrity of group allocation. The primary outcomes of this study were LMA insertion conditions, time to eyelash reflex loss, and time to successful LMA insertion, while the secondary outcomes included haemodynamic response, apnoea duration, pharyngolaryngeal morbidity, and incidence of complications.

### 4.3. Anaesthetic Management

Patients brought to the operating room received standard cardiorespiratory monitoring (electrocardiography (ECG-derivation II), non-invasive blood pressure every minute; systolic arterial pressure (SAP), diastolic arterial pressure (DAP), mean arterial pressure (MAP), pulse oximetry; peripheral oxygen saturation (SpO2), respiratory rate, end-tidal CO2, and inspired oxygen concentration). The depth of anaesthesia was monitored using the bispectral index (BIS) monitor (BIS-Vista™; Aspect Medical Systems, Newton, MA, USA) [17,23]. Patients had venous access opened in the back of the hand with a 20 G cannula, and 7 mL.kg^−1^ saline infusion was administered before induction [16,18,20,21]. Patients were randomly assigned to receive 0.5, 1, 2, or 3 μg.kg^−1^ remifentanil (Groups I, II, III, and IV, respectively; *n* = 25 in each group) by means of individually prepared envelopes. The coded test syringes were prepared by an independent anaesthesiologist to a total volume of 50 mL with 0.9% saline. The anaesthesiologist inserting the mask and monitoring parameters was blind to the drug doses, so injection of all syringes was performed behind a cover by an assistant. Preparation and administration of drugs were performed by a different anaesthesiologist than the one inserting the mask and monitoring parameters. In this way, the anaesthesiologist inserting the mask, observing patient response to LMA insertion, and monitoring and recording parameters was blind to the drugs administered. [16,20]. Nearly three minutes before induction, all patients underwent preoxygenation with 6 L/min oxygen via face mask [45] and premedication with 0.03 mg.kg^−1^ IV midazolam (Dormicum^®^ ampoule, Roche Pharmaceuticals, Istanbul, Turkey), followed by the administration of the study drugs [7,13].

Remifentanil was infused over 60 s with an infusion pump (Braun Infusomat^®^; Braun Melsungen Ko, Melsungen, Germany) [4,21]. Thirty seconds after starting remifentanil, 5 mg.kg^−1^ thiopental (Pental sodium^®^, I.E Ulagay Pharmaceutical Industry, Istanbul, Turkey) was administered over 30 s [10,12,14,23]. When the patient became unconscious, as judged by a loss of response to commands and loss of eyelash reflex, manual ventilation of the lungs was started using a facemask with 100% oxygen [16,19]. Time to eyelash reflex loss (loss of eyelash reflex duration: LED) was determined and recorded as the time from beginning administration of the induction agent to the moment that eyelash reflex was lost [9,17,29]. LMA size was determined based on patient body weight, as recommended in the manufacturer’s guideline, and the same type of mask (Classic) was used for all patients in the study [41]. The mask was lubricated with a water-soluble gel, and the cuffs were fully deflated. Ninety seconds after thiopental administration [16,19,21], patients with a BIS value below 40 [17] and sufficient jaw relaxation had the mask inserted by a single researcher with more than 3 years of experience, using the standard method described by Brain [8,13,40,41]. Criteria showing successful placement of the laryngeal mask were seeing a square wave on the capnogram, easy ventilation with the respiration balloon, seeing chest movements, and not hearing any air leak with a maximum 20 cmH_2_O positive-pressure ventilation [40,46].

The duration of successful insertion (ID: measured from mouth opening to the first successful ventilation) was recorded [40]. After inserting the laryngeal mask, cuff pressure monitoring was performed to standardise postoperative pharyngeal morbidity (cuff pressure manometer, Rüsch, Almanya). The laryngeal mask cuff was inflated until the air in the cuff reached 20–30 cmH_2_O and was maintained below 40 cmH_2_O throughout the operation (cuff pressure manometer, Rüsch, Germany) [47]. Maintenance of anaesthesia used a 50% O_2_/50% N_2_O mix containing 1.5–2% sevoflurane [22]. Sevoflurane concentration was set to keep the BIS value between 40 and 60 [17]. If sufficient induction could not be achieved in patients, if any movements were observed during the first attempt, and if it was necessary to keep BIS values under 40, an additional dose of 2.5 mg.kg^−1^ thiopental was administered; and, 60 s later, a second attempt was made to insert the mask [17,41].The number of attempts was recorded; however, LMA insertion conditions were only evaluated during the first attempt [5,39,40,45]. If there was failure of two attempts during insertion (adequate ventilation could not be provided, hypercarbia (end-tidal CO_2_ > 45) or hypoxemia (SpO_2_ < 95%), sound of a leak, and lack of resolution of these problems with position change), endotracheal intubation was performed. Patients with failure of LMA insertion after two attempts, with an SPO_2_ value below 90% in any period of the study or developing full laryngospasm, were excluded from the study [14,17,41,45,46,47]. LMA insertion conditions were assessed with a six-variable scale used in previous studies [17]. During LMA insertion, a rating system based on chin opening, ease of insertion, swallowing, coughing and gagging, laryngospasm, and patient movement was used, and the tolerance of patients to LMA insertion was assessed. LMA insertion conditions were classified into three groups: excellent—all criteria were excellent; satisfactory—all criteria a mix of satisfactory and excellent; and poor—one or more of the criteria were poor (Table 6).

In all four groups, SAP, DAP, MAP, HR, BIS, and SpO_2_ values were measured at baseline; 1 min before LMA insertion; and in the 1st, 2nd, 3rd, 4th, and 5th minutes after insertion. Apnoea duration (AD: duration between the last spontaneous respiration after induction and the onset of the first spontaneous respiration) was also recorded [17,23]. Sevoflurane was ended 5 min before the end of the operation, and 100% O_2_ was administered. The mask was removed when sufficient ventilation was achieved, and the duration of LMA use (use duration: UD) was recorded (UD: duration between insertion and removal) [48]. After removing the laryngeal mask, the presence of blood was assessed as 1, no blood seen; 2, trace amounts; and 3, a significant amount of blood [49]. The waking patient was taken to the recovery unit with 100% oxygen. To determine the incidence and severity of pharyngolaryngeal complications, a single researcher, blind to group assignment and not included in the anaesthesia process, evaluated all patients on discharge from the recovery unit for throat pain (constant pain, independent of swallowing) and dysphagia (difficulty with swallowing provoked by drinking). Patients were questioned about the presence/absence of these symptoms in the postoperative period. Throat pain was assessed as follows: 0 points for no complaints, 1 point for mild throat pain, 2 points for moderate throat pain, and 3 points for severe throat pain [48]. In the perioperative period, if hypotension developed (MAP < 30% reduction compared to basal values), 6 mg ephedrine (Efedrin, Haver, Istanbul, Turkey) was administered. Bradycardia was defined as HR below 50 beats/min, and 0.5 mg IV atropine (Atropine Sulfate, Haver, İstanbul, Turkey) was administered [18,21].

### 4.4. Ethical Statement

This study was conducted in compliance with the Declaration of Helsinki, approved by the Okmeydani Education and Research Hospital Medication Research Local Ethics Committee (decision number: 110), and registered with the Australian New Zealand Clinical Trials Registry (ACTRN12625000175471). All patients provided written informed consent prior to participation in the study, acknowledging their understanding of the study’s purpose, procedures, and potential risks.

### 4.5. Statistical Analysis

Descriptive statistics (mean, standard deviation, median, minimum, and maximum) are used to describe continuous variables. The distribution of categorical variables is presented using frequencies and percentages. The normality of the data distribution was assessed using the Shapiro–Wilk test and Kolmogorov–Smirnov test. The relationship between more than two independent continuous variables that did not follow a normal distribution was analysed using the Kruskal–Wallis test. For parameters with significant results, post hoc pairwise comparisons were conducted using the Bonferroni-corrected Mann–Whitney U test. The relationship between categorical variables was examined using the Chi-Square test (or Yates’ Continuity Correction/Fisher’s Exact test, where appropriate). The time effect in the groups was examined with a repeated measures ANOVA test. A *p*-value of less than 0.05 was considered statistically significant. Data were evaluated using the IBM SPSS Statistics 26.0 (IBM Corp., Armonk, NY, USA) statistical package program.

### 4.6. Power Analysis

The power analysis for this study was conducted using G Power 3.1.9.6. The effect size for the comparisons of “Excellent LMA insertion” was assumed to be “large”. To achieve a statistical power of 80% with a Type I error rate (α) of 0.05, the minimum sample size to be included in the study was calculated as 52, with a 10% missing value rate, so a minimum of 57 patients were calculated for sample size.

## 5. Conclusions

In conclusion, our study revealed that administration of a single bolus dose of 5 mg.kg^−1^ thiopental for induction, along with 2 µg.kg^−1^ remifentanil, resulted in 96% rates of perfect or satisfactory laryngeal mask airway (LMA) insertion conditions and more stable haemodynamics, without an increase in the duration of apnoea. Based on these findings, 2 µg.kg^−1^ remifentanil is recommended over 3 µg.kg^−1^ for LMA insertion due to its superior balance of efficacy and reduced haemodynamic side effects. This dose provides optimal LMA insertion conditions with fewer risks, including reduced hypotension and shorter apnoea duration, making it the preferred choice for clinical practice.

## Figures and Tables

**Table 1:** Demographic data.

			Group I (*n* = 25)			Group II (*n* = 25)			Group III (*n* = 25)			Group IV (*n* = 25)			*p*-Value
Age (yr)		Mean ± SD	39.68	±	12.58	33.16	±	13.44	35.28	±	11.53	36.40	±	8.42	0.162 ^K^
		Median (min–max)	39.0 (20–65)			29.0 (19–60)			33.0 (20–64)			36.0 (18–51)			
Sex	Female	n-%	12		48.0	13		52.0	12		48.0%	12		48.0	0.989 ^X^2^^
	Male	n-%	13		52.0	12		48.0	13		52.0%	13		52.0	
Weight (kg)		Mean ± SD	70.12	±	11.00	70.20	±	11.93	71.88	±	10.99	76.52	±	13.40	0.194 ^A^
		Median (min–max)	70.0 (50–88)			70.0 (50–95)			72.0 (50–94)			76.0 (54–105)			

^K^ Kruskal–Wallis test/^X^2^^ Pearson’s Chi-squared test/^A^ one-way Anova.

**Table 2:** Induction parameters.

		Group I (*n* = 25)	Group II (*n* = 25)	Group III (*n* = 25)	Group III (*n* = 25)	*p*-Value
LED (s)	Mean ± SD	38.23 ± 7 ^c,d^	35.92 ± 5.48 ^c,d^	31.33 ± 4.9 ^a,b^	28.92 ± 4.43 ^a,b^	<0.001 ^A^
	Median (min–max)	38.0 (27–50)	35.0 (27–45)	30.5 (24–41)	29.0 (22–38)	
AD (s)	Mean ± SD	176.14 ± 77.4 ^c,d^	214.42 ± 72.19 ^d^	232.33 ± 57.74 ^a–d^	346.48 ± 72.94 ^a–c^	<0.001 ^K^
	Median (min–max)	150 (100–466)	193 (123–355)	219 (142–345)	340 (256–505)	
ID (s)	Mean ± SD	18.64 ± 5.99 ^c,d^	15.92 ± 4.21 ^c,d^	12.71 ± 2.24 ^a,b^	11.92 ± 2.18 ^a,b^	<0.001 ^A^
	Median (min–max)	17.5 (10–30)	15.5 (10–25)	13.0 (9–16)	12.0 (8–16)	
UD (min)	Mean ± SD	59 ± 20.25	59.58 ± 27.3	61.96 ± 28.9	63.88 ± 28.42	0.916 ^K^
	Median (min–max)	60.0 (32–107)	59.0 (32–122)	52.0 (32–123)	50.0 (34–126)	

^A^ One-way Anova. ^K^ Kruskal–Wallis test (Bonferroni correction for multiple tests with Mann–Whitney U test). Superscripts indicate the difference between measurements in the same group. ^a^ Different from Group I. ^b^ Different from Group II. ^c^ Different from Group III. ^d^ Different from Group IV. LMA insertion duration = ID; apnoea duration = AD; LMA use duration = UD; loss of eyelash reflex duration = LED.

**Table 3:** Laryngeal mask airway insertion conditions data.

		Group I (*n* = 25)		Group II (*n* = 25)		Group III (*n* = 25)		Group IV (*n* = 25)		*p*-Value
LMA insertion conditions										<0.001 ^X^2^^
Excellent	n %	7	28.0 ^c,d^	10	40.0 ^c,d^	18	72.0 ^a,b^	20	80.0 ^a,b^	
Satisfactory	n %	10	40.0	12	48.0	6	24.0	5	20.0	
Poor	n %	8	32.0	3	12.0	1	4.0	0	0.0	
LMA ease of insertion										0.046 ^F^
Easy	n %	17	68.0 ^c,d^	20	80.0	23	92.0 ^a^	24	96.0 ^a^	
Difficult	n %	5	20.0	4	16.0	1	4.0	1	4.0	
Impossible	n %	3	12.0	1	4.0	1	4.0	0	0.0	
Mouth opening										
Full	n %	12	48.0 ^c,d^	14	56.0 ^c,d^	21	84.0 ^a,b^	22	88.0 ^a,b^	0.003 ^X^2^^
Partial	n %	12	48.0	11	44.0	4	16.0	3	12.0	
Nil	n %	1	4.0	0	0.0	0	0.0	0	0.0	
Swallowing										0.224 ^F^
Nil	n %	19	76.0	22	88.0	23	92.0	24	96.0	
Slight	n %	4	16.0	3	12.0	2	8.0	1	4.0	
Gross	n %	2	8.0	0	0.0	0	0.0	0	0.0	
Coughing–gagging										<0.001 ^X^2^^
Nil	n %	11	44.0 ^b–d^	19	76.0 ^a,c,d^	24	96.0 ^a,b^	25	100.0 ^a,b^	
Slight	n %	8	32.0	4	16.0	1	4.0	0	0.0	
Gross	n %	6	24.0	2	8.0	0	0.0	0	0.0	
Head and limb movement										0.502 ^F^
Nil	n %	22	88.0	23	92.0	24	96.0	25	100.0	
Slight	n %	2	8.0	2	8.0	1	4.0	0	0.0	
Gross	n %	1	4.0	0	0.0	0	0.0	0	0.0	
Laryngospasm										0.057 ^F^
Nil	n %	22	88.0	25	100.0	25	100.0	25	100.0	
Slight	n %	3	12.0	0	0.0	0	0.0	0	0.0	
Gross	n %	0	0.0	0	0.0	0	0.0	0	0.0	
Number of attempts										0.075 ^F^
I	n %	16	64.0	21	84.0	22	88.0	23	92.0	
II	n %	6	24.0	3	12.0	2	8.0	2	8.0	
III	n %	3	12.0	1	4.0	1	4.0	0	0.0	

^X^2^^ Pearson’s Chi-squared test. ^F^ Fisher’s exact test. Superscripts indicate the difference between measurements in the same group. ^a^ Different from Group I. ^b^ Different from Group II. ^c^ Different from Group III. ^d^ Different from Group IV.

**Table 4:** Haemodynamic parameters (median).

		Group I (*n* = 25)	Group II (*n* = 25)	Group III (*n* = 25)	Group IV (*n* = 25)	*p*
Heart Rate (bpm)						
Basal	Mean ± SD	80.32 ± 10.21	80.5 ± 13.7	84.68 ± 12.6	85.32 ± 11.9	0.317 ^A^
	Median (min–max)	82 (62–105)	80 (50–102)	85 (57–105)	86 (65–112)	
1 min before LMA	Mean ± SD	84.48 ± 11.11 ^d,^*	77.2 ± 12.9 *	78.08 ± 12.48 *	73.76 ± 11.31 ^a,^*	0.019 ^A^
	Median (min–max)	85 (64–109)	76 (50–103)	80 (53–96)	73 (55–97)	
1 min after LMA	Mean ± SD	86.76 ± 11.48 ^c,d,^*	78.1 ± 13.2 *	76.24 ± 11.42 ^a,^*	73.12 ± 11.07 ^a,^*	0.001 ^A^
	Median (min–max)	86 (66–114)	78 (52–101)	77 (54–92)	73 (54–99)	
2 min	Mean ± SD	85.28 ± 11.36 ^c,d^*	75.6 ± 12.8 *	74.52 ± 11.35 ^a,^*	71.88 ± 10.97 ^a,^*	0.001 ^A^
	Median (min–max)	85 (65–113)	75 (51–100)	75 (52–90)	71 (52–97)	
3 min	Mean ± SD	82.84 ± 11.4 ^c,d^	73.2 ± 12.2 *	72.32 ± 11.58 ^a,^*	70.64 ± 10.71 ^a,^*	0.001 ^A^
	Median (min–max)	84 (63–110)	73 (50–95)	74 (50–89)	70 (53–95)	
4 min	Mean ± SD	79.88 ± 11.43 ^b–d^	70.3 ± 11.1 ^a,^*	71.16 ± 11.85 *	70.16 ± 10.49 ^a,^*	0.006 ^A^
	Median (min–max)	81 (60–106)	69 (50–92)	74 (49–89)	70 (53–95)	
5 min	Mean ± SD	78.56 ± 10.79 ^b–d^	69 ± 11.0 ^a,^*	70.08 ± 11.4 *	69.44 ± 10.75 ^a,^*	0.007 ^A^
	Median (min–max)	79 (60–103)	69 (49–90)	71 (50–88)	69 (52–93)	
Mean arterial pressure (mmHg)						
Basal	Mean ± SD	91.92 ± 9.48	93.28 ± 10.95	95.44 ± 9.5	97.92 ± 11.25	0.169 ^K^
	(min–max)	93 (77–114)	91 (73–117)	92 (82–115)	97 (78–127)	
1 min before LMA	Mean ± SD	87.76 ± 9.89 ^d,^*	84.32 ± 9.79 ^d,^*	83.64 ± 7.28 ^d,^*	75.88 ± 6.69 ^a–c,^*	<0.001 ^A^
	Median (min–max)	87 (72–107)	82 (66–105)	82 (72–100)	75 (65–90)	
1 min after LMA	Mean ± SD	89.04 ± 9.63 ^d,^*	84.84 ± 9.39 ^d,^*	83.56 ± 6.77 ^d,^*	74.68 ± 4.77 ^a–c,^*	<0.001 ^A^
	Median (min–max)	89 (75–109)	83 (68–106)	82 (72–97)	75 (67–84)	
2 min	Mean ± SD	86.68 ± 9.2 ^d,^*	83.16 ± 8.79 ^d,^*	82.08 ± 7.5 ^d,^*	73.56 ± 5.08 ^a–c,^*	<0.001 ^A^
	Median (min–max)	86 (71–105)	81 (68–101)	81 (69–97)	73 (66–83)	
3 min	Mean ± SD	84.64 ± 8.94 ^d,^*	80.72 ± 9.01 ^d,^*	79.92 ± 7.09 ^d,^*	71.6 ± 5.12 ^a–c,^*	<0.001 ^A^
	Median (min–max)	84 (68–102)	79 (65–99)	79 (68–95)	72 (64–82)	
4 min	Mean ± SD	82.36 ± 8.83 ^d,^*	79.12 ± 8.66 ^d,^*	77.88 ± 7.14 ^d,^*	70.8 ± 4.94 ^a–c,^*	<0.001 ^A^
	Median (min–max)	82 (66–99)	77 (65–96)	77 (67–94)	71 (64–81)	
5 min	Mean ± SD	81.36 ± 8.63 ^d,^*	78.56 ± 8.78 ^d,^*	77.04 ± 7.3 ^d,^*	69.88 ± 4.91 ^a–c,^*	<0.001 ^K^
	Median (min–max)	82 (67–98)	77 (63–96)	75 (66–93)	70 (64–81)	

^A^ One-way Anova. ^K^ Kruskal–Wallis test (Bonferroni correction for multiple tests with Mann–Whitney U Test). Superscripts indicate the difference between measurements in the same group. ^a^ Different from Group I. ^b^ Different from Group II. ^c^ Different from Group III. ^d^ Different from Group IV. Repeated measures ANOVA group effect, *p* < 0.001; time effect, *p* < 0.001; group *p* < 0.001. * Different from Basale.

**Table 5:** Postoperative complication.

			Group I (*n* = 25)		Group II (*n* = 25)		Group III (*n* = 25)		Group IV (*n* = 25)		*p*-Value
**Presence of blood**	1	n %	17	77.3	20	83.3	22	91.7	24	96	*0.219* ^X^2^^
	2	n %	3	13.6	3	12.5	2	8.3	1	4	
	3	n %	2	9.1	1	4.2	0	0	0	0	
**Recovery sore throat**	0	n %	15	68.2	18	75	20	83.3	22	88	0.363 ^X^2^^
	1	n %	2	9.1	2	8.3	2	8.3	2	8	
	2	n %	2	9.1	2	8.3	2	8.3	1	4	
	3	n %	2	9.1	2	8.3	0	0	0	0	
	4	n %	1	4.5	0	0	0	0	0	0	
**Recovery dysphagia**	No	n %	16	72.7	20	83.3	22	91.7	23	92	*0.249* ^X^2^^
	Yes	n %	6	27.3	4	16.7	2	8.3	2	8	

^X^2^^ Pearson’s Chi-squared test.

**Table 6:** Laryngeal mask airway insertion score.

	Insertion Conditions		
**Variable**	Excellent	Good	Poor
Mouth opening	Full	Partial	Nil
Ease of LMA insertion	Easy	Difficult	Impossible
**Patient responses**			
Swallowing	Nil	Slight	Gross
Coughing/gagging	Nil	Slight	Gross
Head and body movement	Nil	Slight	Gross
Laryngospasm	Nil	Slight	Gross

LMA insertion conditions: excellent, all responses are excellent; satisfactory, all responses are excellent or satisfactory; poor, the presence of one or more poor responses.

## Data Availability

The data presented in this study are available upon request from the corresponding author due to (privacy, legal, or ethical reasons).
